# Dysfunction of IKZF1/MYC/MDIG axis contributes to liver cancer progression through regulating H3K9me3/p21 activity

**DOI:** 10.1038/cddis.2017.165

**Published:** 2017-05-04

**Authors:** Qi Huo, Chao Ge, Hua Tian, Ji Sun, Meiling Cui, Hong Li, Fangyu Zhao, Taoyang Chen, Haiyang Xie, Ying Cui, Ming Yao, Jinjun Li

**Affiliations:** 1 State Key Laboratory of Oncogenes and Related Genes, Shanghai Cancer Institute, Renji Hospital, Shanghai Jiaotong University School of Medicine, Shanghai, China; 2 Qi Dong Liver Cancer Institute, Qi Dong, China; 3 Department of General Surgery, The First Affiliated Hospital, School of Medicine, Zhejiang University, Hangzhou, China; 4 Cancer Institute of Guangxi, Nanning, China

## Abstract

MDIG is known to be overexpressed in many types of human cancers and has demonstrated predictive power in the prognosis of cancer, although the functions and mechanisms of MDIG in liver cancer, especially in hepatocellular carcinoma (HCC), are still unknown. In this study, we report that MDIG and MYC were negatively regulated by IKZF1. MDIG overexpression substantially promoted HCC cell proliferation, cell migration and spreading, whereas knockdown of MDIG would reverse above-mentioned effect. MDIG effects on tumour cell growth were further demonstrated in a tumour xenograft model. Moreover, MDIG had effects on the level of p21(CIP1/WAF1) via H3K9me3 expression in HCC. MDIG was also found to be closely related to the sorafenib resistance of HCC cells *in vitro*. Clinically, we found that MDIG was frequently overexpressed in human HCCs (69.7% *n*=155) and was significantly associated with histological grade and hepatitis B virus infection. Our findings indicate that MDIG plays an important role in HCC progression via MDIG/H3K9me3/p21(CIP1/WAF1) signalling and serves as a potential therapeutic target.

Hepatocellular carcinoma (HCC), which is the dominant form of primary liver cancer, is a complex liver disease that constitutes a major global health problem. HCC has a high mortality rate and is the third leading cause of cancer-related deaths worldwide; it accounts for more than half a million deaths per year.^1, 2^ Despite current developments in clinical therapies, including surgical resection, radiotherapy and chemotherapy, the high mortality rate of patients with HCC and their poor prognosis are attributed in part to the difficulty in obtaining a diagnosis at an early stage.^3, 4, 5^ Upregulation of certain genes has been shown to promote hepatocarcinogenesis and may even play a role in the protection of tumour cells against clinical treatment.^6, 7^ Furthermore, the development of tumours and the potential for metastasis are frequently associated with alterations in gene expression profiles. Therefore, the identification of potential biological markers for early-stage diagnosis and novel therapeutic strategies are the focus of molecular targeted therapy in patients with HCC to improve their prognosis.^8, 9, 10^

As with most cancers, HCC does not result from a single genetic change; instead, it results from the upregulation of a series of oncogenes.^7, 8^ MDIG is a protein with a molecular weight of 53 kDa that contains a conserved JmjC domain and is primarily localized in the nucleus. The expression of MDIG may play a key role in cell proliferation.^11, 12, 13, 14, 15^ Several reports suggest that MDIG is overexpressed in a variety of human cancers,^16, 17, 18, 19, 20^ including glioblastoma,^21^ oesophageal squamous cell carcinoma,^22^ colon cancer,^23^ lung cancer,^24^ gastric cancer^25, 26^ and cholangiocarcinoma.^27, 28^ In addition, the forced expression of MDIG in NIH/3T3 cells induces cell transformation and produces tumours in nude mice,^29^ whereas the targeted silencing of MDIG delays cell cycle progression and inhibits cell proliferation. These data show that MDIG has oncogenic potential; therefore, this protein has been implicated in carcinogenesis.

To elucidate the functional role of MDIG in HCC, we found that overexpression of MDIG promotes tumour cell growth, migration, and spreading and suppresses the level of H3K9 trimethylation in HCC. MDIG regulates the expression of p21(CIP1/WAF1) via H3K9 demethylation. In addition, we found that MDIG was upregulated in HCC tissues and was significantly associated with histological grade. MDIG was also found to be related to the sorafenib resistance of HCC cells. We explored MDIG inhibition by the overexpression of the transcription factor IKZF1. These findings suggest that MDIG may serve as a valuable biomarker for early-stage diagnosis in HCC. Furthermore, MDIG may be a novel therapeutic target in the treatment of patients with HCC.

## Results

### Effects of IKZF1 expression on MDIG and MYC in HCC cells

**Figure 1:**
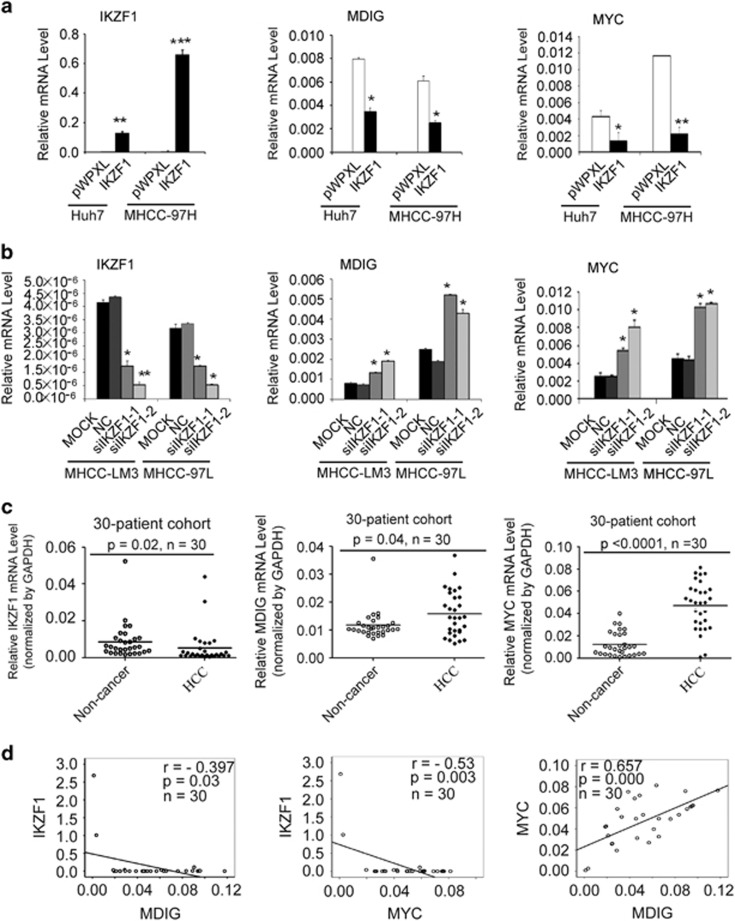
The expression of IKZF1 was negatively associated to those of MYC and MDIG in HCC. (**a**) qRT-PCR analysis showed that reduced MDIG and MYC mRNA expression (middle and right panel) in Huh7 and MHCC-97H cells after IKZF1 ectopic expression (left panel). GAPDH was used for normalization control. The graph is shown as mean±s.d. of *n*=3 independent experiments. Unpaired Student’s *t*-tests were used for statistical analysis (*P<0.05, **P<0.01, ***P<0.001). (**b**) Effect of IKZF1 silencing (left panel) on MDIG and MYC mRNA levels (middle and right panel) in MHCC-LM3 and MHCC-97 L cells were determined by qRT-PCR and normalized to GAPDH. The graph is shown as mean±s.d. of *n*=3 independent experiments. Unpaired Student’s *t*-tests were used for statistical analysis (*P<0.05, **P<0.01). (**c**) mRNA levels of IKZF1(left panel), MDIG (middle panel) and MYC (right panel) in a cohort of 30 pairs of human primary HCC patients; HCC, cancer tissue; non-cancer, matched adjacent non-cancerous liver tissues. The data were normalized to GAPDH. Unpaired *t*-tests were used for statistical analysis (mean±s.d., *n*=30, *P<0.05, ***P<0.001). (**d**) The correlation of IKZF1, MDIG and MYC mRNA expression was analysed in 30 primary human HCCs. Pearson’s correlation coefficient (*r*) and statistical significance are indicated. *P<0.05, **P<0.01, ***P<0.001

We examined whether MDIG mRNA and protein levels were affected by IKZF1 in HCC cells. Following ectopic expression of IKZF1 in Huh7 and MHCC-97H cells, MDIG and IKZF1 mRNA levels were measured by qRT-PCR. MDIG mRNA levels were reduced at a comparable level in IKZF1-overexpressed cells, compared with vector control cells (Figure 1a). In contrast, following silence of IKZF1 expression in MHCC-LM3 and MHCC-97L cell lines, qRT-PCR revealed a ∼1.84- and ∼ 2.27-fold rise in MDIG mRNA levels, compared to negative controls, respectively (Figure 1b). Consistently, the expression levels of MYC were repressed by IKZF1 in HCC cells (Figures 1a and b). Furthermore, western blot analysis confirmed that overexpression of IKZF1 in Huh7 and MHCC-97H cell lines decreased MDIG and MYC protein levels (Supplementary Figure 1a), whereas knockdown of IKZF1 in MHCC-LM3 and MHCC-97L cell lines increased MDIG and MYC protein levels (Supplementary Figure 1b). These results suggested that the expression levels of MDIG and MYC, in fact, were affected by IKZF1 in HCC cells.

### Negative correlation of MDIG and MYC levels and IKZF1 expression in HCC

From our *in vitro* results, mRNA expression of MDIG was associated with the mRNA level of IKZF1. To further clarify the relation between MDIG and IKZF1, relative mRNA levels of MDIG and IKZF1 were examined by way of qRT-PCR in 30 human HCC tissues. HCCs exhibited underexpression of IKZF1 mRNA levels compared with their corresponding non-tumorous livers (Figure 1c); however, MDIG mRNA was expressed at high levels in HCC samples compared with the matched non-cancer liver tissue (Figure 1c), and a similar result was obtained in a TCGA cohort (Supplementary Figure 1c). This drastic decrease in mRNA levels of IKZF1 in the tumours may not imply a signature change in HCC, but a potential role of this change in the elevation of MDIG mRNA. Based on these data, we further evaluated possible associations between MDIG and IKZF1 mRNA in human HCCs. Pearson’s correlation analysis, which was used to examine the correlation between MDIG and IKZF1 mRNA in HCC tumour tissues, yielded a correlation of -0.397, with a *P*-value of 0.03 (Figure 1d). MYC mRNA was expressed at higher levels in HCC samples compared with the matched non-cancer liver tissue (Figure 1c). Furthermore, MYC mRNA levels were negatively correlated to that of IKZF1 in human HCCs (Figure 1d). These results indicated that underexpression of IKZF1 was significantly associated with MDIG overexpression in human HCCs.

### MDIG and MYC gene promoter activity are inhibited by the transcription factor IKZF1 in HCC cell lines

**Figure 2:**
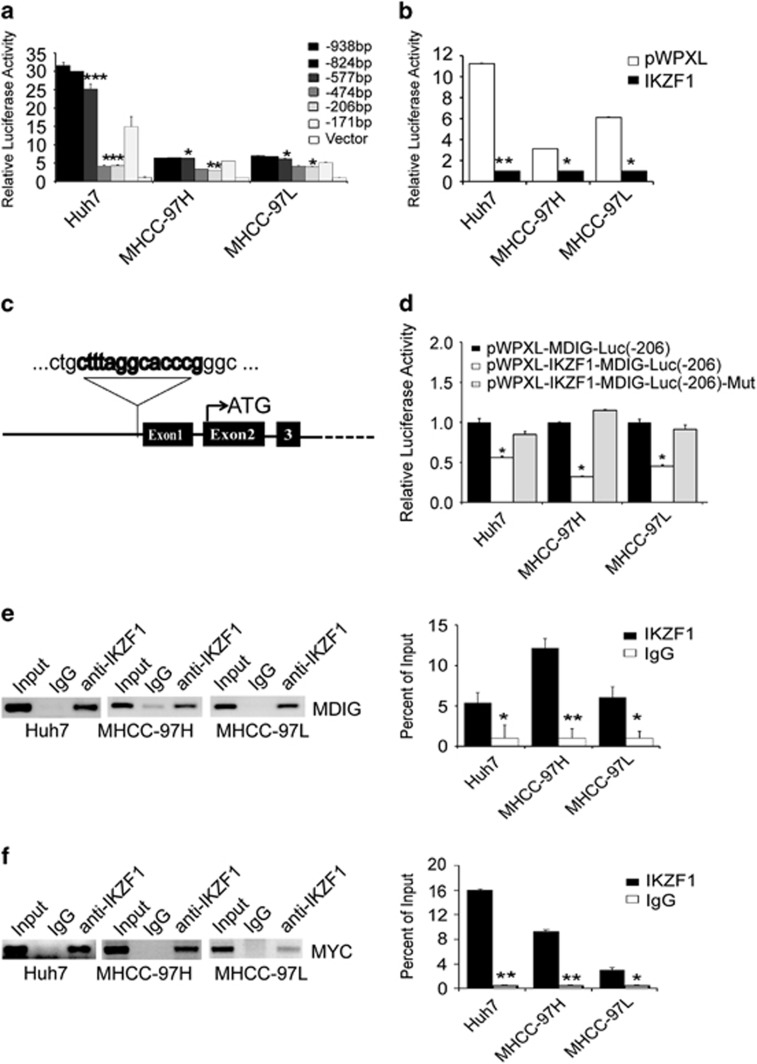
IKZF1 directly regulates the expression of MDIG and MYC in HCC cells. (**a**) Various lengths of human MDIG gene promoter plasmids containing the luciferase gene were constructed and transfected into Huh7, MHCC-97H and MHCC-97L cells. After 24 h, luciferase activities were measured and normalized to the activity of the pGL3 basic vector (Vector) transfected into cells. The negative regulatory element is localized in the -206/-171 bp region. (**b**) The promoter activities in the -206 bp region were inhibited after transfection of Huh7, MHCC-97H and MHCC-97L cells with IKZF1. (**c**) Putative transcription factor IKZF1 binding sites on the MDIG promoter are indicated. (**d**) The plasmid pWPXL-IKZF1 and the MDIG luciferase reporter vectors (wild-type or mutant IKZF1 binding sites, in the -206/-171 bp region) were co-transfected into Huh7, MHCC-97H and MHCC-97L cells. The relative luciferase activities were determined by a reporter gene assay. IKZF1 rescued MDIG promoter activity when the predicted binding site was mutated. (**e**, **f**) CHIP analysis showed increased binding of IKZF1, respectively, with MDIG (**e**) and MYC (**f**) promoter region, rabbit IgG was used as negative control. Quantitative analysis of CHIP data was performed on the bound fraction, and IgG was used as a negative control. The graph of (**a**), (**b**), (**c**), (**d**), (**e**) and (**f**) are shown as mean±s.d. of *n*=3 independent experiments. Unpaired Student’s *t*-tests were used for statistical analysis (*P<0.05, **P<0.01, ***P<0.001)

Because of this negative correlation between MDIG and IKZF1, we hypothesized that IKZF1, as a transcription factor, may directly regulate MDIG gene expression. To this end, our further experimental efforts were focused on determining whether MDIG is a direct target of IKZF1. We initially investigated human MDIG promoter activity using a series of 5′-promoter deletion analysis. Deleted promoter fragments were ligated into a dual luciferase reporter gene vector pGL3 basic and were transiently transfected into Huh7, MHCC-97H and MHCC-97L cell lines. Relative luciferase activities of deleted promoter constructs are shown in Figure 2a, suggesting that a putative positive cis-element is localized in the -577/-474 bp region. In addition, a negative regulatory element is localized in the -206/-171 bp region. Therefore, the promoter region of MDIG has binding sites for both enhancers and silencers of transcription. Next, we found that IKZF1 reduced MDIG promoter activity when p-206 was assayed in Huh7, MHCC-97H and MHCC-97L cell lines (Figure 2b). However, IKZF1 failed to reduce MDIG promoter activity when the predicted binding site for IKZF1 was mutated (Figures 2c and d). To gain further insight into the mechanism of reduction, chromatin immunoprecipitation (CHIP) assays and their quantification results clearly confirmed that IKZF1 was directly bound to MDIG promoter in Huh7, MHCC-97H and MHCC-97L cell lines (Figure 2e). Then, we also performed CHIP assays and their quantification results showed IKZF1 was directly bound to MYC promoter in HCC cell lines (Figure 2f). As expected, these experiment results in our study clearly demonstrated that IKZF1 affects MDIG expression by directly binding to MDIG promoter, which confirmed that MDIG is a *bona fide* target of IKZF1.

### Stable expression of MDIG promotes tumour cell growth

**Figure 3:**
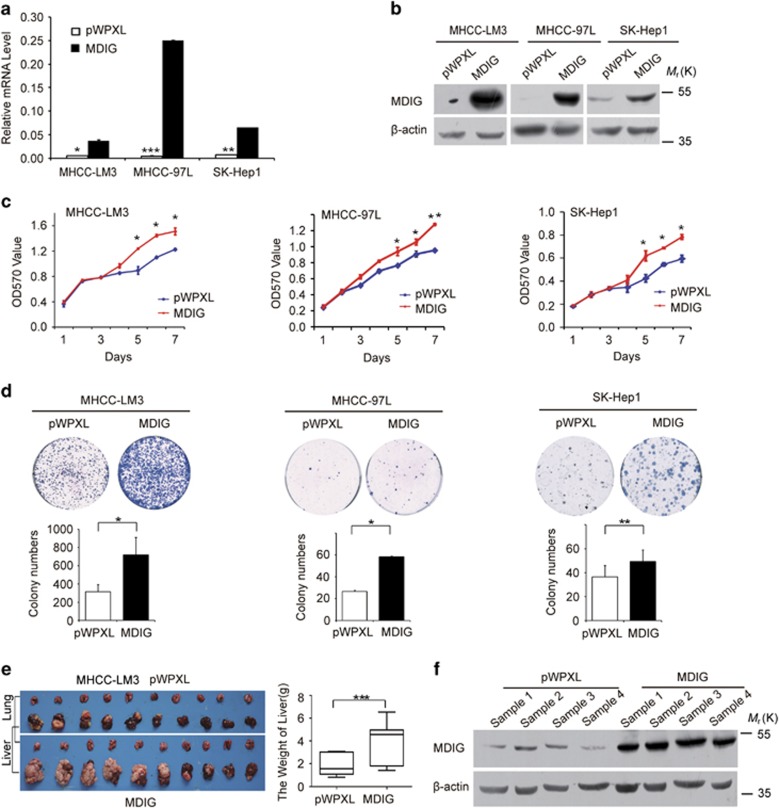
Ectopic expression of MDIG promotes HCC cell proliferation and tumorigenicity *in vitro* and *in vivo*. (**a**,**b**) MDIG mRNA (**a**) and its protein (**b**) level were detected, respectively, by qRT-PCR and by western blot in MHCC-LM3, MHCC-97L and SK-Hep1 cells after MDIG forced expression with lentiviral vector (pWPXL) *in vitro*. (**c**) Ectopic expression of MDIG promotes the proliferation of HCC cells by MTT assay. (**d**) MDIG promotes the colony formation ability of HCC cells, and bar graphs showed their quantitative analysis data. (**e**) Liver and lung tissues from the animals with tumour xenografts inoculated with MHCC-LM3 cell line stably expressing MDIG, and bar graphs showed their quantitative analysis of the liver weights. (**f**) MDIG protein level was detected by western blot in xenografts. The graph of (**a**), (**c**), (**d**) and (**e**) are shown as mean±s.d. of *n*=3 independent experiments. Unpaired Student’s *t*-tests were used for statistical analysis (*P<0.05, **P<0.01, ***P<0.001)

To investigate the biological effects of MDIG in HCC, we addressed the question whether overexpression of MDIG affects tumour cell growth *in vitro*. First, MDIG mRNA and protein in HCC cell lines were, respectively, analysed by qRT-PCR and western blot. We found that the MHCC-LM3, MHCC-97L and SK-Hep1 cell lines normally express low endogenous levels of MDIG mRNA and protein (Supplementary Figures 2a and b). We successfully established three HCC cell lines with stable overexpression of MDIG clones (Figures 3a and b), which were then used to study the effects of this protein on proliferation of HCC cells. Here, stable overexpression of MDIG significantly promoted cell proliferation in three HCC cell lines, compared to vector controls (Figure 3c). To further observe the effects of MDIG on HCC cell growth, their colony formation ability was assessed. After 2 weeks of growth, a clear trend towards an increase in both the number and size of the colonies was revealed. MDIG expression augmented the colony formation ability of these cells as evidenced by the increase in the size and number of colonies formed. Specifically, a 1.5 ∼2-fold increase was observed in the number and size of the colonies in MDIG-overexpressed HCC cell lines (Figure 3d). Based on these results, our proliferation assay and colony formation ability assay suggested that MDIG promotes tumour cell growth *in vitro*.

We next addressed the question whether overexpression of MDIG promotes tumour cell growth *in vivo*. To answer this question, we performed tumour xenograft *in situ* liver experiments with MHCC-LM3 cells stably overexpressing MDIG as well as vector control cells using immunodeficient nude mice. Thirty-five days later, we observed the overall weight of liver and average volume of tumour at the time of killing were significantly increased in xenografts that overexpressed MDIG (*n*=10 mice per group; Figure 3e). Moreover, we found that MDIG-overexpressing mice indeed exhibited high levels of MDIG in xenograft tissues (Figure 3f). Our *in vivo* data were consistent with the *in vitro* data and strengthened the biological importance of MDIG in HCC.

### Silence of MDIG inhibits tumour cell growth *in vitro* and *in vivo*

**Figure 4:**
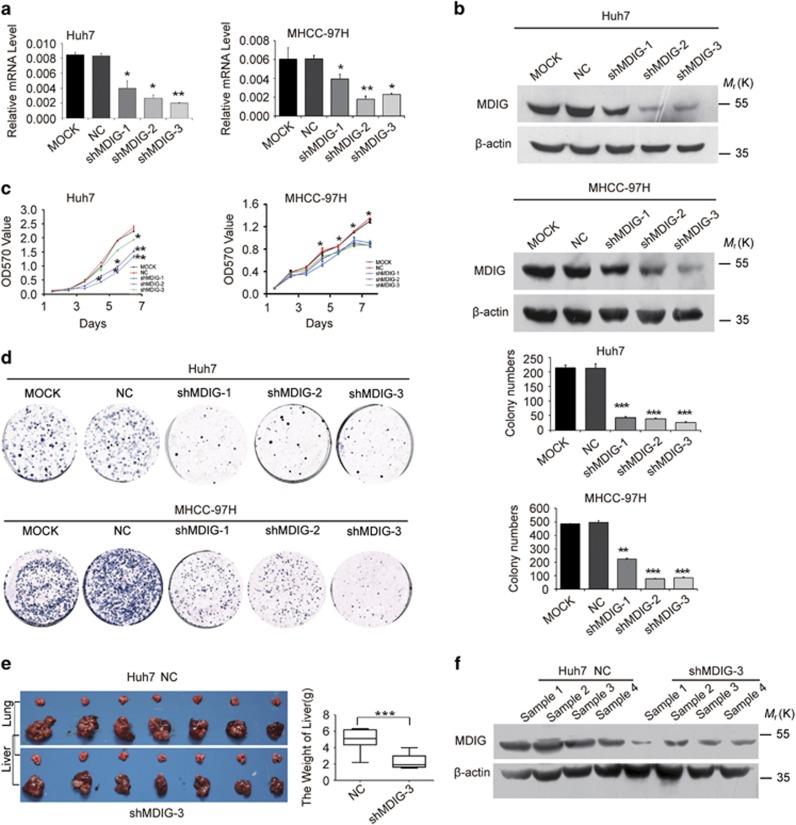
Knockdown of MDIG inhibits tumour cell growth *in vitro* and *in vivo*. (**a**, **b**) MDIG mRNA (**a**) and its protein (**b**) level were detected, respectively, by qRT-PCR and by western blot in Huh7 and MHCC-97H cells as a quality control after MDIG knockdown (shMDIG) with shRNA *in vitro*. MOCK and NC were, respectively, served as blank and negative control. (**c**) Silencing expression of MDIG (shMDIG) decreases the cell growth of HCC cells by MTT assay. (**d**) Silencing expression of MDIG (shMDIG-1, -2, -3) remarkably reduced the colony formation ability of HCC cells, and bar graphs showed their quantitative analysis data. (**e**) Liver and lung tissues from the animals with tumour xenografts inoculated with Huh7 cell line stably expressing shMDIG-3, NC as a negative control; and bar graphs showed their quantitative analysis of the liver weights. (**f**) MDIG protein level was detected by western blot in Huh7 cell xenografts of shMDIG. The graph of (**a**), (**c**), (**d**) and (**e**) are shown as mean±s.d. of *n*=3 independent experiments. Unpaired Student’s *t*-tests were used for statistical analysis (*P<0.05, **P<0.01, ***P<0.001)

Given that MDIG was associated with tumour cell proliferation *in vitro* and *in vivo*, we examined whether knockdown of MDIG in HCC cells would inhibit their growth abilities. We previously observed that MDIG was differentially expressed across a panel of HCC cell lines. By lentiviral transduction of shRNA, we established stable knockdown MDIG clones in HCC cell lines (Huh7 and MHCC-97H), which have high MDIG expression (Figures 4a and b). Antitumour effects of MDIG shRNA treatment were seen in Huh7 and MHCC-97H cells, in which cell proliferation was suppressed, compared with negative control (Figure 4c). In addition, knockdown of MDIG expression remarkably inhibited the colony formation ability of these cells, and after 2 weeks of growth, a decrease in the number and size of the colonies was seen (Figure 4d).

Thus, we next explored the antigrowth effect of MDIG knockdown *in vivo* by orthotopic xenograft model. MDIG-specific shRNA (Huh7-shMDIG-3) and scrambled shRNA cells (Huh7-NC) were inoculated into nude mice. At 7 weeks after tumour cell inoculation, large tumours were seen in the control groups, but the size of the tumours was still minimal in the mice that were given MDIG shRNA (Figure 4e). Moreover, tumours in animals with cells expressing MDIG-specific shRNA reduced MDIG level, compared to control group (Figure 4f). These findings suggested that knockdown of MDIG could reverse the effect of it overexpression *in vitro* and *in vivo*.

### MDIG promotes migration and invasion of HCC cells *in vitro*

**Figure 5:**
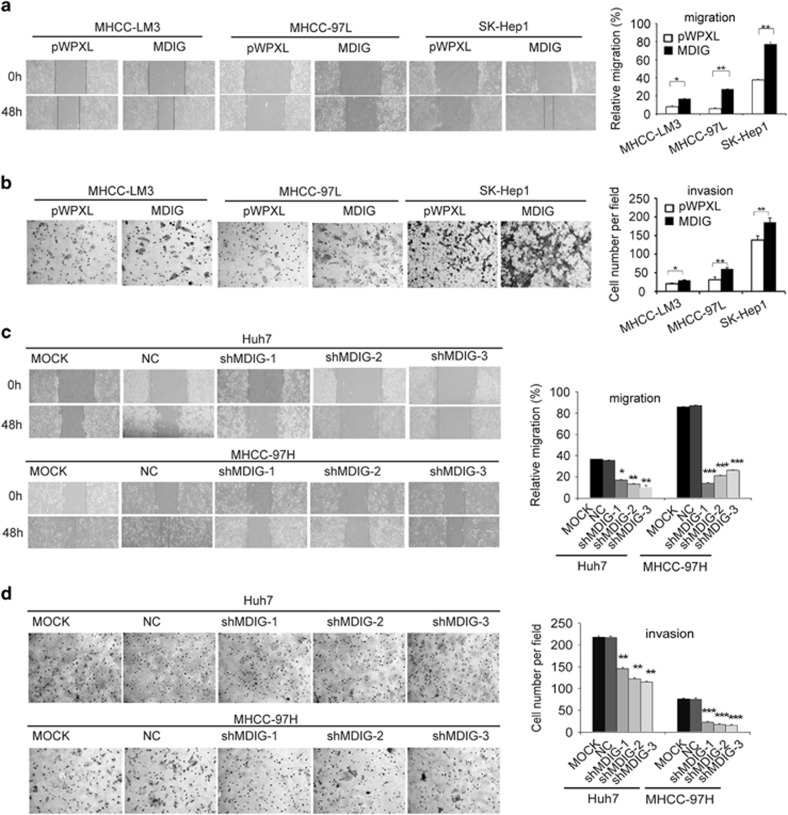
MDIG promotes migration and invasion of HCC cells. (**a**) Stably overexpressed MDIG with lentiviral vector (pWPXL) promotes *in vitro* migration of MHCC-LM3, MHCC-97L and SK-Hep1 cells. Quantitative data are presented (original magnification: × 100). (**b**) Ectopic MDIG expression promotes *in vitro* invasion of MHCC-LM3, MHCC-97L and SK-Hep1 cells. Quantitative results are presented (original magnification: × 200). (**c**) Knockdown of MDIG (shMDIG-1, -2, -3) suppresses *in vitro* migration in Huh7 and MHCC-97H cells compared to negative control (NC) and blank cell control (MOCK). Quantitative results are shown (original magnification: × 100). (**d**) Knockdown of MDIG suppresses *in vitro* invasion of Huh7 and MHCC-97H cells. Quantitative data are shown (original magnification: × 200). The graph of (**a**), (**b**), (**c**) and (**d**) are shown as mean±s.d. of *n*=3 independent experiments. Unpaired Student’s *t*-tests were used for statistical analysis (*P<0.05, **P<0.01, ***P<0.001)

To further investigate other biological effects of MDIG in HCC, we performed a migration assay with MHCC-LM3, MHCC-97L and SK-Hep1 cells stably overexpressing MDIG. We observed that migration was increased in MDIG overexpressing cells, compared to control cells (Figure 5a). Furthermore, we used a Transwell invasion assay with Matrigel to analyse the invasiveness of HCC cells, which overexpress MDIG. MDIG overexpression could dramatically promote the invasion ability of HCC cells (Figure 5b). We were next interested in whether inhibition of MDIG gene expression affects migration and invasion of HCC cells. We knocked down the expression of MDIG by shRNA in the HCC cell lines Huh7 and MHCC-97H, which have relatively high levels of MDIG. We observed that knockdown of MDIG in HCC cells significantly inhibited migration and invasion, respectively, in both Huh7 and MHCC-97H cells (Figures 5c and d). These results indicated that MDIG promotes the migration and invasion behaviour of HCC cells.

### Effects of MDIG expression on histone H3K9 methylation

**Figure 6:**
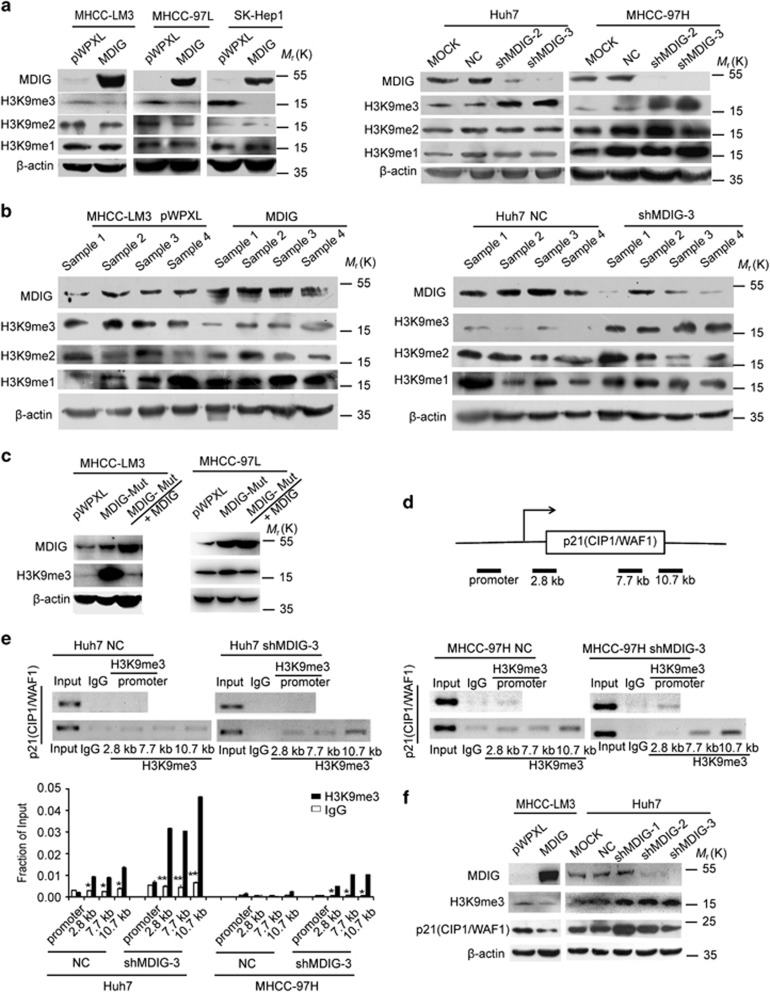
MDIG regulates expression of p21(CIP1/WAF1) via H3K9 demethylation. (**a**) Western blot analysis of MDIG and H3K9me3 status in MHCC-LM3, MHCC-97L and SK-Hep1 cells stably transfected with an empty vector (pWPXL) or MDIG (left panel). MDIG and H3K9me3 status in Huh7 and MHCC-97H cells stably transfected with blank control (MOCK) and negative control shRNA (NC) or MDIG shRNA (right panel). (**b**) Western blot analysis of MDIG and H3K9me3 status of the xenograft tissues with ectopic expression MDIG in MHCC-LM3 cell (left panel) and with MDIG knockdown in Huh7 cells (right panel). (**c**) Western blotting of H3K9me3 in MHCC-LM3 and MHCC-97L cells after stable expression of spot MDIG mutant, and then stable overexpression of MDIG, compared to controls. Mut, mutant. (**d**,**e**) CHIP analysis showed increased binding of H3K9me3 and p21(CIP1/WAF1) in Huh7 and MHCC-97H knockdown MDIG cells; the positions of primers for promoter and transcript regions of p21(CIP1/WAF1) are indicated (**d**). CHIP (top panel) and quantitative analysis of CHIP data (bottom panel) were performed on the bound fraction in Huh7 and MHCC-97H cells (**e**), and rabbit IgG was used as an isotype control. The graph of (**e**) is shown as mean±s.d. of *n*=3 independent experiments. Unpaired Student’s *t*-tests were used for statistical analysis (*P<0.05, **P<0.01, ***P<0.001). (**f**) Western blotting of H3K9me3 and p21(CIP1/WAF1) in MHCC-LM3 MDIG overexpressing cells or Huh7 MDIG knockdown cells. *P<0.05, **P<0.01, ***P<0.001

We next want to understand the role of MDIG in HCC cells. MHCC-LM3, MHCC-97L and SK-Hep1 cell lines were stably transfected with the MDIG gene. The levels of MDIG and H3K9me3 were detected by western blot, in which ectopic expression of MDIG remarkably decreased the levels of H3K9me3 (Figure 6a). Conversely, knockdown of MDIG by lentiviral shRNA significantly increased H3K9me3 levels in Huh7 and MHCC-97H cell lines (Figure 6a). However, no distinct alteration of H3K9me2 and H3K9me1 levels were observed in MDIG-overexpressing or MDIG-knockdown HCC cell lines. In line with these observations, we also found that MDIG could affect the expression of H3K9me3 in xenograft tissues (Figure 6b). Taken together, our data implicated that H3K9me3 downregulation is facilitated by overexpression of MDIG in HCC.

### MDIG regulates expression of p21(CIP1/WAF1) via H3K9 demethylation

To further confirm that MDIG has a demethylation activity on H3K9me3, we designed experiments to determine whether the alteration of H3K9me3 is related with MDIG-demethylation activity. We constructed an MDIG-mutant plasmid (MDIG contains a mutation at lysine 194) and analysed expression of H3K9me3. As shown in Figure 6c and Supplementary Figure 3a, with the increase in MDIG caused by overexpression of MDIG-mutant in MHCC-LM3 and MHCC-97L cells, the level of H3K9me3 showed a significant increase. Subsequently, on overexpressing MDIG in MHCC-LM3 and MHCC-97L cells with MDIG-mutant stably expressed and level of H3K9me3 got a notable decline. The data indicate that MDIG has a demethylation activity on H3K9me3 and the mutation of demethylation activity disabled this ability. To identify the functions for H3K9me3 in HCC cells, we analysed by the CHIP and qPCR the distribution of H3K9me3 regulated p21(CIP1/WAF1) gene. H3K9me3 has not been detected primarily at repressed promoter regions of p21(CIP1/WAF1) gene (Figures 6d and e). Unexpectedly, the data showed H3K9me3 associated with the transcribed regions and activated the p21(CIP1/WAF1) gene (Figures 6d and e). Western blotting showed that knockdown of MDIG resulted in a substantial increase in H3K9me3 and p21(CIP1/WAF1) expression in Huh7 cells (Figure 6f). On overexpressing MDIG in MHCC-LM3 cells, a reversed phenomenon was observed (Figure 6f). Based on these results, we considered that MDIG regulates expression of p21(CIP1/WAF1) via H3K9 demethylation and H3K9me3 associated with the transcribed regions and activated the p21(CIP1/WAF1) gene.

### MDIG remarkably alters the sensitivity of sorafenib to HCC cells *in vitro*

To understand how the MDIG affects the drug resistance of HCC cells, we examined the growth inhibitory effect of sorafenib in HCC cell lines. First, ectopic expression of MDIG in MHCC-LM3, MHCC-97L and SK-Hep1 cells could decrease the sensitivity of HCC cells to various doses of sorafenib, compared to those of the control cells infected with the pWPXL vector (Supplementary Figure 4a). Sorafenib inhibited cell growth in these three cell lines at a 50% inhibitory concentration (IC_50_). The IC_50_ values were increased, compared to vector control (Supplementary Figure 4b). Then, silencing MDIG expression in HCC cells remarkably enhanced the sensitivity of Huh7 and MHCC-97H cells to sorafenib (Supplementary Figure 4c). Sorafenib suppressed MDIG-knockdown HCC cell growth in a dose-dependent manner, and its IC_50_ was distinctly decreased (Supplementary Figure 4d). These results indicated that silencing MDIG sensitizes the cells to sorafenib’s growth inhibition.

### Differential expressions of MDIG, MYC, H3K9me3 and p21(CIP1/WAF1) in HCC patients and clinical significance analysis

**Figure 7:**
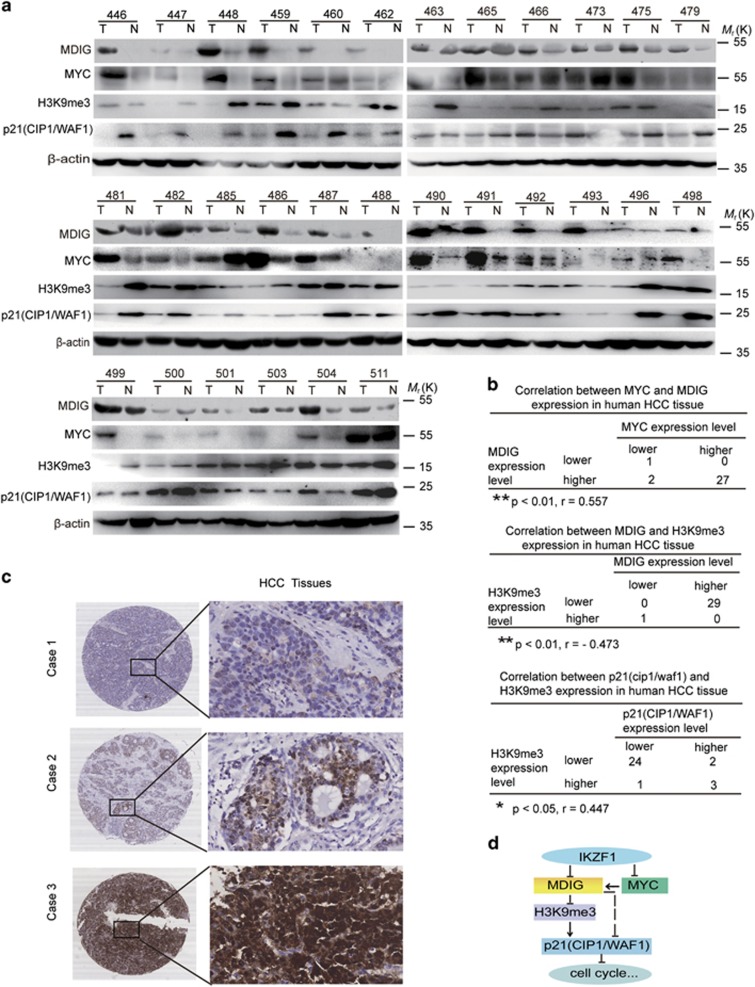
Higher expression levels of MDIG with HCC patients. (**a**) Analysis of MDIG, MYC, H3K9me3 and p21(CIP1/WAF1) expression in human primary HCC and adjacent non-cancerous tissues by western blot. T, HCC tissue; N, matched adjacent non-cancerous liver tissue. *β*-actin was used as an internal loading control. (**b**) Several correlation analyses of MDIG, MYC, H3K9me3 and p21(CIP1/WAF1) protein levels in 30 human primary HCC and matched adjacent non-cancerous liver tissues were presented. Pearson’s chi squared test was used; *r*, correlation coefficient. (**c**) IHC analysis of MDIG protein in HCC samples. Cases 1, 2 and 3, respectively, represent MDIG protein weakly, moderately and strongly stained in HCC tissues; original magnification for right panel: × 400). (**d**) The regulation model of MDIG in HCC. MDIG is regulated by IKZF1 and MYC; MDIG is able to downregulate the expression level of H3K9me3, which is bound to the transcribed regions of p21(CIP1/WAF1), and further activated it. *P<0.05, **P<0.01, ***P<0.001

Based on our above data and to further understand the important role of MDIG and qualify the expression of MDIG in HCC patients, western blot analysis was applied to detect MDIG expression in clinical HCC specimens and the paired adjacent non-tumorous livers from 30 patients. The relative expression of MDIG was higher in most HCC tissues compared with their matched non-cancerous liver tissues (Figure 7a). These data collectively showed that MDIG overexpression was frequently seen in HCC patients. Because expression of IKZF1 was detected by western blot in our previous data,^30^ we examined the MYC, H3K9me3 and p21(CIP1/WAF1) expression in clinical HCC tissue samples (Figure 7a). A negative correlation between the expression levels of MDIG and H3K9me3, a reversed phenomenon was observed in the expression levels of MYC and MDIG (Figure 7b). Furthermore, a positive correlation between the expression levels of H3K9me3 and p21(CIP1/WAF1) was observed in our cohort (Figure 7b). According to the IHC results (Figure 7c), the expression intensity of MDIG was scored as 0 or 1, for weak and strong immunostaining, respectively. MDIG protein overexpression was frequently (69.7%, 108 of 155) observed in HCCs. As to clinicopathological correlation, MDIG overexpression was significantly associated with the histological grade (*P*=0.024, Table 1), hepatitis B surface antigen (HBsAg) levels (*P*=0.001), hepatitis B e antigen (HBeAg) levels (*P*=0.042, Table 1), and hepatitis B core antibody (HBcAb) levels (*P*=0.001, Table 1). Overall survival analysis revealed that overexpression of MDIG in HCCs was closely associated with poorer overall survival rates, although it did not reach statistical significance (Supplementary Figure 5a).

Overall, our findings showed that MDIG could promote HCC cell growth, migration, and spreading and drug resistance, and itself regulated by IKZF1 and MYC. Moreover, MDIG is able to downregulate the expression level of H3K9me3, whereas H3K9me3 directly bound to the transcribed regions of p21(CIP1/WAF1) and activated its expression. Therefore, MDIG, regulated by IKZF1 and MYC, which decreases H3K9me3/p21(CIP1/WAF1) signalling and further affects the HCC progression (Figure 7d).

## Discussion

MDIG has attracted intense research interest because of its implication in the regulation of cell proliferation and survival.^21, 22, 26, 28^ We focused on the expression of MDIG using two experimental strategies *in vitro*: (1) After the induction of stable MDIG expression by a lentiviral expression system, we observed its effects on cell growth, colony formation ability, migration and spreading. The forced expression of MDIG promotes HCC cell growth, colony formation, migration and spreading *in vitro*. (2) When MDIG was inhibited by stable RNA interference, the opposite effects were observed. The knockdown of MDIG inhibits tumour cell growth, colony formation, migration and spreading *in vitro*. Similar results of tumour cell growth were found *in vivo* in our tumour xenograft animal models. Currently, accumulating evidence clearly indicates the involvement of MDIG in mammalian cell proliferation and metastasis. Here, we provide evidence of the correlation between high MDIG expression and HCC tumorigenesis, and demonstrate the oncogenic properties of MDIG in a genetically defined mouse model. Overexpression of MDIG is positively correlated with metastatic potential in HCC cell lines. Additionally, other studies have shown that MDIG is frequently expressed in human cancers. For instance, overexpression of MDIG is associated with high metastatic potential, positive lymph node metastasis and shorter Overall Survival (OS) in gastric cancer patients.^25, 26^ However, a paradoxical effect of MDIG on cell growth and motility at different stages of neoplastic transformation in non-small cell lung cancer has also been suggested.^31^ MDIG overexpression was also correlated with poorer overall survival of lung cancer patients, especially those without lymph node metastasis. Moreover, MDIG expression was higher in poorly differentiated HCC tissues, and significantly related to the histological grade as observed by IHC.^28^ Based on our results, MDIG expression is associated with hepatitis B virus infection. MDIG serves as an attractive therapeutic target for HCC because it is highly expressed in tumour tissues but not in healthy liver tissues. Most of HCC (69.7% *n*=155) samples are positive for MDIG. Thus, the difference in the expression pattern of MDIG between HCC and lung cancer may be context-dependent or may be due to different regulatory mechanisms.^32, 33, 34, 35^

Our previous study confirmed that IKZF1 plays an important role in HCC.^30^ Additionally, IKZF1 bound to the regulatory regions of MYC and suppressed MYC expression in acute lymphoblastic leukemia cells.^36^ In the current study, we show that in HCC cell lines, MDIG levels are decreased by overexpression of IKZF1. As a repressor of transcription, IKZF1 may regulate the expression of MDIG. To demonstrate that MDIG is a direct target gene of IKZF1, we examined the activity of the MDIG promoter. Furthermore, we showed that the suppression of MDIG expression is directly due to the activity of the transcription factor IKZF1. We also identified one of the sites to which IKZF1 binds directly, and we functionally validated this binding site by mutation experiments. Indeed, mutations in the binding site completely abolished the suppression of the MDIG reporter by IKZF1. The lack of a DNA binding domain led to the inability to repress MDIG, which indicates that IKZF1 directly represses MDIG.

H3K9me3, as a key epigenetic marker, and its misregulation has been observed in many human cancers.^37, 38, 39, 40^ MDIG contains a conserved JmjC domain, which is a motif served by histone demethylation. Our results implicated that MDIG dereased the level of H3K9me3 in HCC cells. Because H3K9me3 is also tightly associated with the instability of genome,^41, 42^ it would be interesting to further explore the novel mechanism for H3K9me3 in HCC. We herein report that H3K9me3 associated with the transcribed regions and activated the p21(CIP1/WAF1) gene.

Our present study shows that the targeted interference of MDIG either alone or in combination with the overexpression of IKZF1 can inhibit the growth, migration and spread of HCC cells. Clinically, because of acquired resistance after sorafenib treatment, which greatly limits its therapeutic efficacy in HCC patients,^43, 44^ we found and confirmed in this study that the decreased expression of MDIG may be a selective manner to increase the sensitivity of HCC cells to sorafenib, the mechanism of which needs to be further clarified. Taken together, all of these data undoubtedly indicate that as an oncogene MDIG plays an important role in tumour cell growth, migration, and spreading and that MDIG may serve as a suitable target for cancer prevention or therapy.

## Materials and Methods

### Cell culture and reagents

HCC cell lines used in this study include SK-Hep1 (from the American Type Culture Collection, USA), Huh7 (from the Health Science Research Resources Bank, Japan), and MHCC-97H, MHCC-97L and MHCC-LM3 (from Liver Cancer Institute, Zhongshan Hospital of Fudan University, Shanghai, China). Human embryonic kidney 293 T cell lines were purchased from the American Type Culture Collection. All the cell lines were cultured in Dulbecco’s Modifed Eagle Medium (DMEM, Gibco, New York, USA). The medium was supplemented with 10% fetal bovine serum (FBS, Gibco, New York, USA) and 100 units/ml penicillin (Sigma-Aldrich, St. Louis, MO, USA) plus 100 μg/ml streptomycin (Sigma-Aldrich). All of cells were cultured in a 37°C incubator with 5% CO_2_ and a humidified atmosphere.

### Luciferase activity assay

Luciferase activity was assessed according to the dual-luciferase reporter assay protocol (Promega, Madison, WI, USA). Huh7, MHCC-97H and MHCC-97L cells were seeded in 96-well plates at 75% confluence. After 12 h, the cells were transfected with pGL3-Promoter-UTR or were cotransfected with pGL3-Promoter-UTR and pWPXL-IKZF1 using Lipofectamine 2000 (Invitrogen, Carlsbad, CA, USA). Twenty-four hours after transfection, the cells were harvested for the firefly and Renilla luciferase activity assay. The Renilla luciferase activities were used to normalize the transfection efficiency. Following primer sequences were used for cloning: human MDIG (-938)-F (5′-CGACGCGTGGATTGACTC TCTCTCCCGG-3′), human MDIG (-824)-F (5′-CGACGCGTCTCCCTAAACTTGCACCGC-3′), human MDIG (-577)-F (5′-CGACGCGTCTGGGCGAGAGTGAGACCT-3′), human MDIG (-473)-F (5′-CGACGCGTGGACTAAAGAAACGCCCGG-3′), human MDIG(-206) -F(5′-CGACGCGTCGCTGCTGCTTTAGGCAC-3′), human MDIG (-171) -F (5′-CGACGCGTTCTGGGAGACCTGTCGGTC-3′), and human MDIG (universal) -R (5′-GGAAGATCTCCACGTGTGCGACTCTCTG-3′); human MDIG-mutant-F (5′-CGACGCGTCGCTGCTGATCTATTCAACATGGCTCGGGTGCGGGTCTG-3′) and human MDIG-mutant-R (5′-CCCAGGTCCCGCAACCACAG-3′).

### Chromatin immunoprecipitation assay

The CHIP assay was performed using Huh7, MHCC-97H and MHCC-97L cells. The cells were cross-linked with 10% paraformaldehyde for 10 min at 37 °C, followed by reversal of the crosslinks with 1 M glycine for 5 min at 37 °C. The cells were harvested in Tissue Protein Extraction Reagent (Thermo Scientific, MA, USA) for 5 min on ice after washing with phosphate-buffered saline (PBS) and centrifugation at 2000 rpm for 5 min. The precipitates were then suspended in nuclei lysis buffer, and the DNA was shredded into fragments of 1000 base pairs by sonication. Antibodies against IKZF1 (Santa Cruz Biotechnology, Texas, USA) on protein G agarose beads (Sigma-Aldrich) were added and incubated overnight at 4 °C. DNA was then isolated and used for polymerase chain reaction (PCR) analysis. Assays were conducted in duplicates in three independent experiments. The primers used for the quantitative PCR are provided as follows: human MDIG-F (5′-AACAGG CCGTGCCGGGTG-3′), human MDIG-R (5′-CCCAGGTCCCGCAACCACA G-3′); human MYC-F (5′-AGGGGAAGGGAGGGGAAGGGAAAG-3′), human MYC-R (5′-GAGAACAGTTGAAACACAA-3′); human p21(CIP1/WAF1)-promoter-F (5′-GAAAGCAGAGGGGCTTCAAG-3′), human p21(CIP1/WAF1)-promoter-R (5′-TGTCTGCACCTTCGCTCCT-3′);human p21(CIP1/WAF1)-2.8 kb-F (5′-GGTTCAGGTCCTTTACGCCACT-3′), human p21(CIP1/WAF1)-2.8 kb-R (5′-TCACAGTGAGTCACCTCCTCGC-3′); human p21(CIP1/WAF1)-7.7 kb-F (5′-ACTGTGATGCGCTAATGGCG-3′), human p21(CIP1/WAF1)-7.7 kb-R (5′-AGGCGAAGTCACCCTCCAGT-3′); human p21(CIP1/WAF1)-10.7 kb-F (5′-CCATGTGTCCTGGTTCCCGT-3′), human p21(CIP1/WAF1)-10.7 kb-R (5′-AGCATTGTGGGAGGAGCTGTG-3′).

### Vector construction and lentivirus infection

The open read frame cDNA sequences of human MDIG and IKZF1 were amplified, and subcloned into the lentivirus expression vector pWPXL, and we successfully obtained the pWPXL-MDIG and pWPXL-IKZF1 fusion expression clones. The primer sequences were used for cloning as follows: human MDIG-F: 5′-CGACGCGTATGCCAAAGAAAGCAAAGC-3′, human MDIG-R: 5′-CGACGCGT ATGCCAAAGAAAGCAAAGC-3′ human IKZF1-F: 5′-ATTGGATCCATGGATGCTGATGAGGGTC-3′, human IKZF1-R: 5′-ATTGAATTCTTAGCTCATGTGGAAGC-3′. All constructs were verified by sequencing. HEK-293 T cells were transfected with pWPXL-MDIG and pWPXL-IKZF1 using Lipofectamine 2000 (Invitrogen), along with the packaging and envelope plasmids psPAX2 and pMD2.G (Addgene, Cambridge, MA, USA) according to the manufacturer’s protocol. Virus particles were harvested 48 h after transfection. The HCC cells were infected with recombinant lentivirus using polybrene (Sigma-Aldrich). pLVTHM-shMDIG vector with the packaging plasmid psPAX2 and the envelope plasmid pMD2.G (Addgene) were transfected using Lipofectamine 2000 (Invitrogen). Recombinant lentivirus was produced by transient transfection of HEK-293 T cells. Huh7 and MHCC-97H cells were infected with recombinant lentivirus. Small interfering RNA fragment sequences for shMDIG and NC are listed as follows: human shMDIG-1 (5′-GCCUUCAGCUUUAAACUUUTT-3′), human shMDIG-2 (5′-GGCUCGAAUGUGUACAUAATT-3′), human shMDIG-3 (5′-GGUGGAAUCCACAACUGUUTT-3′) and NC (5′-UUCUCCGAACGUGUCACGUTT-3′).

### RNA interference

The small interfering RNA was designed to target IKZF1 and was synthesized by GenePharma (Shanghai, China) and was transfected with Lipofectamine 2000 (Invitrogen) according to the manufacturer’s instructions. All transfections were performed in serum-free/antibiotic-free media. The silencing effects of sequences were analysed by western blot. Target sequences information is listed as follows: human siIKZF1-1 (5′-GUCGUGGCCAGUAAUGUUATT-3′) and human siIKZF1-2 (5′-GACGCACUCCGUUGGUAAATT-3′).

### RNA extraction and real-time quantitative polymerase chain reaction

Total RNA of snap frozen tissue specimens and cell lines were extracted by using TRIzol reagent (Invitrogen) according to the manufacturer’s protocol. The purity and concentration of RNA were determined by absorbance at 260 nm using a NanoDrop ND-2000 spectrophotometer (NanoDrop Technologies, Wilmington, NC, USA). Reverse transcription was performed on 1 μg of total RNA using M-MLV reverse transcriptase (Promega) according to the manufacturer’s instructions. Newly synthesized cDNA was amplified by qRT-PCR to detect the expression levels. The relative expression levels were calculated by normalization against GAPDH. qRT-PCR was performed using SYBR Green Master Mix with an Applied Biosystems 7500 Software v2.0.5 real-time PCR system (Thermo Scientific). The thermal profile consisted of an initial denaturation step at 95 °C for 10 min, followed by 40 cycles at 95 °C for 30 s, and a final step at 60 °C for 1 min. The melting curve was determined at 95 °C for 15 s, 60 °C for 15 s and 72 °C for 15 s to confirm the specificities of the resulting products. Data were analysed using the comparative threshold cycle method. All experiments were performed in triplicates. The following primer sequences were used for qRT-PCRs: human MDIG-F:5′-CTGCTCCTGCAGGTGGAAT-3′, human MDIG-R: 5′-CCCATCTCCCGCAGAGTAA-3′ human IKZF1-F: 5′-ATGGGCGTGCCTGTGAAATGA-3′, human IKZF1-R: 5′-GCCGTTCTCCAGTGTGGCTTCTT-3′ human MYC-F: 5′-CCTTCTCTCCGTCCTCGGAT-3′, human MYC-R: 5′-TTCTTGTTCCTCCTCAGAGTCG-3′ human GAPDH-F: 5′-AGAAGGCTGGGGCTCATTTG-3′, human GAPDH-R: 5′-AGGGGCCATCCACAGTCTTC-3′.

### Protein extraction and western blot

All snap frozen tissue specimens and cells were lysed by RIPA buffer (Thermo Scientific) containing a protease inhibitor cocktail (Roche, Welwyn Garden, Swiss, UK). Samples were centrifuged at 12 000 rpm for 15 min at 4 °C, and protein concentrations were quantified using the bicinchoninic acid method (Sigma-Aldrich). Thirty micrograms of protein per sample were denatured in loading buffer prior to 10% or 15% SDS-PAGE electrophoreses, and then transferred onto nitrocellulose membranes (Bio-Rad, Hercules, CA, USA). Non-specific binding was blocked by incubating the membranes in 5% non-fat milk for 1 h at room temperature. Membranes were then incubated overnight at 4 °C with either rabbit anti-MDIG (1:200; Abcam, Cambridge, MA, USA, ab155335), rabbit anti-IKZF1 (1:200; Santa Cruz, SC-13039), rabbit anti-H3K9me3 (1:200; Abcam, ab8898), rabbit anti-H3K9me2 (1:200; Abcam, ab115159) and rabbit anti-H3K9me1 (1:500; Abcam, ab194693) primary antibodies. After washing in phosphate-buffered saline-Tween-20 (PBST), the membranes were incubated with horseradish peroxidase-goat anti-rabbit antibody (1:3000; Sigma-Aldrich, A0545) at room temperature for 2 h, then washed again in PBST. Western blots were visualized using enhanced chemiluminescence (ECL) detection reagent with an ECL kit (Cell Signaling Technology, Danvers, MA, USA). Antibodies are shown in Supplementary Table 1.

### Cell proliferation assay

The transfected cells were seeded into 96-well culture plates at a density of 1 × 10^3^ cells per well and incubated for 24 h. Then, 10 μl MTT (50 mg /ml) was added to each well. The media was removed after cells were incubated for 4 h at 37 °C and 100 μl DMSO was added in the dark. The OD was recorded at an absorbance of 570 nm. Each assay was performed in triplicates.

### *In vitro* colony formation assay

The transfected cells were plated in six-well culture plates at a density of 1000 cells per 2 ml well and incubated for 15 days. Then, cells were washed with PBS and fixed in neutral buffered formalin at room temperature for 40 min and stained with Giemsa (Sigma-Aldrich) for another 30 min. The numbers of stained cell clones were counted. Each assay was performed in triplicate.

### *In vitro* invasion assays

The invasion assays were carried out using 24-well transwell chambers with an 8-μm pore polycarbonate membrane insert. The cells were seeded at a density of 2 × 10^5^ on the top side of the membrane in pre-chilled serum-free DMEM with Matrigel and incubated for 48 h. Cells were fixed in neutral buffered formalin and stained with Giemsa (Sigma-Aldrich) and the invasion efficiency was determined by microscopy. Six fields were randomly selected from each chamber. Assays were conducted in duplicates in three independent experiments.

### Sorafenib resistance assay

Cells were cultured into 96-well culture plates and incubated for 24 h, sorafenib (Biochempartner, Shanghai, China) was diluted to final concentrations with culture medium. After being treated with drug for 48 h, 10 μl MTT (50 mg/ml) was added, and the media was removed after cells were incubated for 4 h at 37 °C, and 100 μl DMSO was added in the dark. The OD value was recorded at an absorbance of 570 nm. Each assay was performed in triplicate.

### Tumour xenograft models

For the tumour xenograft assays, 2 × 10^6^ MHCC-LM3 cells infected with pWPXL-MDIG or empty vector and 3 × 10^6^ Huh7 cells infected with shMDIG-3 or negative control (NC) were resuspended in 25 μl of serum-free DMEM and mixed the same volumes of Matrigel (1:1) on ice bath for each *BALB/c-nu/nu* mouse (*n*=10 per group), and injected the cells to murine liver under anesthesia as soon as possible. After 5-7 weeks, the animals were killed and their livers and lungs were removed. The tumour xenografts were weighed and fixed in neutral buffered formalin and prepared for histological examination.

### Patients

HCC specimens collected for qRT-PCR and western blot detection were clinically and histologically diagnosed at the First Affiliated Hospital of Zhejiang University (Hangzhou, China), Guangxi Cancer Institute (Nanning, China) and Qidong Liver Cancer Institute (Qidong, China). All samples were obtained during surgery prior to the administration of radiotherapy or chemotherapy. Informed consent was obtained from each patient, and the study was performed in accordance with the China Ethical Review Committee.

### Immunohistochemistry assay

For immunohistochemistry assays, HCC tissue sections were probed with MDIG polyclonal antibody (Invitrogen) at a dilution of 1:25. Horseradish peroxidase-conjugated secondary antibody was applied at 37 °C for 30 min and incubated with diaminobenzidine solution, and hematoxylin was used for tissue counterstaining. Immunostaining of each HCC case in the tissue microarray was compared to the matched adjacent liver tissue by semiquantitative scores. Immunohistochemical scores were obtained as follows: the intensity of staining was categorized into 0 and 1, denoting weakly stained and strong stained, respectively. Antibodies are shown in Supplementary Table 1.

### Statistical analysis

Statistical analyses were performed using SPSS v19.0 statistical software (SPSS, Chicago, IL, USA). A paired-sample *t*-test was used to evaluate differences in the expression levels of MDIG mRNA and protein between the HCC specimens and the matched adjacent non-tumour tissues. A two-tailed unpaired Student’s *t*-test was used to assess differences in cell proliferation rates, colony formation, cell migration and invasion. Significant differences from at least three independent experiments are expressed as the means±s.d. A two-sided *P*-value<0.05 was considered statistically significant. Pearson’s correlation analysis was used to compare mRNA or protein expression of two genes. A two-sided *P*<0.05 was considered to be significantly different, and the bigger the correlation coefficient (*r*) of them, the closer their expression.

## Figures and Tables

**Table 1:** Clinicopathological features of 155 HCC patients analysed

**Clinicopathological features**	**MDIG**^a^		***P*** **value**
	**Score 0**	**Score 1**	
*Age*			
<60	32	76	0.713
≥60	15	31	
			
*AFP (ng/mL)*			
≤20	17	37	0.880
>20	30	69	
			
*HBsAg*			
Negative	15	12	0.001*
Positive	29	93	
			
*HBsAb*			
Negative	40	97	0.141
Positive	3	2	
			
*HBeAg*			
Negative	34	87	0.128
Positive	9	11	
			
*HBeAb*			
Negative	29	49	0.042*
Positive	14	51	
			
*HBcAb*			
Negative	16	12	0.001*
Positive	27	87	
			
*Tumour size (cm)*			
≤5	19	55	0.126
>5	28	47	
			
*Histological grade*			
I, II	16	58	0.024*
III, IV	31	50	
			
*Intrahepatic metastasis*			
Absent	31	73	0.842
Present	16	35	
			
*Cirrhosis*			
Absent	6	8	0.285
Present	41	100	

Abbreviations: AFP, alpha-fetoprotein; HCC, hepatocellular carcinoma; MDIG, MYC induced nuclear antigen

a HCC case numbers

a *P* value represents the probability from a Chi-square test for different immunohistochemical scores of MDIG in HCC tissues. * *P*<0.05.

Score 0 represents the expression intensity of MDIG was weakly immunostained. Score 1 denotes MDIG expression was strongly stained
